# Impact of intrapartum oxytocin administration on neonatal sucking behavior and breastfeeding

**DOI:** 10.1038/s41598-024-56635-9

**Published:** 2024-03-11

**Authors:** Machiko Omaru, Setsu Kajiwara, Eri Wakamatsu, Sumiko Kuroishi, Yukifumi Ochiai, Kentaro Oniki, Kiyoko Kato, Seiichi Morokuma

**Affiliations:** 1 Department of Health Sciences, Graduate School of Medical Sciences, Kyushu University, Fukuoka, 812-8582 Japan; 2 Department of Nursing, Comprehensive Maternity and Perinatal Care Center, Kyushu University Hospital, Fukuoka, 812-8582 Japan; 3 Research & Development Division, Pigeon Corporation, Tokyo, 103-8480 Japan; 4 Division of Pharmacology and Therapeutics, Graduate School of Pharmaceutical Sciences, Kumamoto University, Kumamoto, 862-0973 Japan; 5 Department of Obstetrics and Gynecology, Graduate School of Medical Sciences, Kyushu University, Fukuoka, 812-8582 Japan

**Keywords:** Neonatology, Paediatric research, Health care, Medical research

## Abstract

This study aimed to examine the effect of intrapartum oxytocin administration on neonatal sucking behavior and breastfeeding. A total of 64 pairs (29 in the group treated with intrapartum oxytocin and 35 in the control group) of normal infants within 24–48 h of birth and their mothers were recruited. Sucking ability was evaluated by measuring Non-Nutritive Sucking (NNS) for 5 min. Data on the rate of exclusive breastfeeding at 1 month postpartum were collected. In the adjusted multiple regression models, intrapartum oxytocin exposure was significantly associated with fewer total NNS bursts (95% confidence interval (CI), −7.02 to −0.22), longer pause times (95% CI, 1.33 to 10.21), and greater pause-time variability (95% CI, 3.63 to 63.92). Effects estimated using structural equation modeling revealed that intrapartum oxytocin exposure had a significant negative and direct effect on the practice of exclusive breastfeeding 1 month postpartum (β = −0.238, *p* = 0.047). However, no NNS-mediated indirect effects were observed. This report demonstrates that infants born to mothers who receive intrapartum oxytocin may have impaired sucking ability for at least the first 48 h after birth, and breastfeeding support should be provided.

## Introduction

The induction and augmentation of labor during delivery have been increasing worldwide. Reports have shown that 25% of all full-term infants born in developed countries were born after induction or augmentation of labor^1–3^. Additionally, in some areas of low- and middle-income countries, the use of oxytocin for labor induction or augmentation exceeded 50% of obstetric care facilities^4^. Oxytocin is a peptide hormone released from the posterior pituitary gland. During labor, endogenous oxytocin is released in pulses from the pituitary glands of women into the peripheral bloodstream to induce regular uterine contractions^5^. Maximum levels of endogenous oxytocin are achieved within 1 h of delivery in both maternal and infant brains. Maternal oxytocin release increases via skin-to-skin contact with the infant^6,7^. This hormone promotes uterine restoration in postpartum mothers and is an essential hormone released during breastfeeding that causes the breast milk ejection reflex^8^.

In cases that require labor induction and augmentation, synthetic oxytocin is the most commonly used uterotonic agent. In contrast to the natural endogenous oxytocin secretion mechanism, the use of continuous intravenous administration of exogenous synthetic oxytocin preparations has been reported to have negative connotations. Specifically, it has been suggested that the administration of exogenous oxytocin disturbs the mechanism of physiological secretion, and the state of excess oxytocin in the mother causes negative feedback and suppresses endogenous oxytocin release^9^. As a result, not only is there a possibility of reduced endogenous oxytocin secretion, but also of suppressed oxytocin receptors^10,11^. Intrapartum oxytocin administration can also disrupt the balance of the endogenous oxytocin secretion in the fetus by allowing exogenous oxytocin to pass through the placental barrier and immature fetal blood–brain barrier, causing negative feedback and negatively affecting postnatal feeding and water metabolism^12^. Animal data have shown that perinatal manipulation of the oxytocin system has lasting effects on the feeding, attachment, sociability, and sexual behavior of children^13^.

Oxytocin is an important hormone for breastfeeding, and there have been reports on the impact of intrapartum synthetic oxytocin administration on breastfeeding^14^. Current reports can be classified into two categories: those that survey the “impact on breastfeeding,” and those that survey the “impact on feeding behavior of infants.” The impact on breastfeeding has been reported to be associated with oxytocin administration, lower early postpartum breastfeeding initiation rates^15,16^, and lower long-term breastfeeding rates^17–19^. However, some reports^20,21^ indicated no association with breastfeeding rates, and certain conclusions have not been reached. Impacts on the feeding behavior of infants have been reported in previous studies on the association between intrapartum oxytocin administration and lower expression of primitive neonatal reflexes^22,23^. Primitive reflexes associated with feeding refers to the instinctive behavior of newborns to seek, find, and begin to suck on the nipple of their mother. In previous studies, newborns were placed skin-to-skin on the breasts of their mothers, and their behavior leading up to spontaneous sucking was filmed using a study-specific scale to identify the primitive reflexes of the newborn, as well as to compare the number of primitive reflexes and the rate of eventual effective sucking with and without oxytocin administration. Reduced primitive reflexes associated with feeding have been observed in studies not only at 1 h after birth but also at 48 h after birth^19,24^. Although synthetic oxytocin preparations are fast falling in blood levels, it has been suggested that prolonged infusion of exogenous oxytocin at delivery may alter the neuroendocrine environment of the fetal brain and that the effect lasts for at least 48 h^19,24^. Zhou et al.^25^. also visually observed primitive reflexes during skin to skin contact in 154 newborn infants within 2 h of birth, divided into maternal delivery oxytocin high-dose, medium-dose, and low-dose groups. Newborns in the high-dose group took longer time to start suckling and had a shorter effective suckling time than those in the control and low-dose groups. It is suggested that the negative effect of oxytocin administration during delivery on neonatal suckling behavior may be drug dose-dependent.

Several methods exist to assess infant feeding performance to ascertain whether maternal administration of oxytocin during delivery affects the infant's feeding behavior. In previous studies, infant feeding behavior was evaluated by visual observation of primitive reflexes through videography^22,23^ . However, new findings are expected to be obtained by conducting studies with more objective parameters. The first method measured sucking during direct breastfeeding, in which infants are fed directly through their mother’s breasts^26^. However, sucking measurements during direct breastfeeding result in differences in the lactation conditions between cases. Specifically, differences in the mother’s inexperience with lactation techniques, speed of breast milk flow into the infant’s oral cavity, and breast and nipple morphology make condition matching difficult. The next method involved measuring sucking during bottle feeding with formula or breast milk^27,28^. However, bottle-feeding infants for research purposes may be a barrier to establishing breastfeeding. Therefore, in this study, we measured the infants’ Non-Nutritive Sucking (NNS) under the same conditions using empty nipples and compared the infants' sucking ability. More recent evidence indicates that negative pressure formation, in which the infant draws in milk through an intraoral vacuum, is more important for effective feeding, than positive pressure, which is applied by the mandible^29^. Therefore, we planned to measure negative pressure in the infant's oral cavity during sucking in this study. Oxytocin affects the motor neurons associated with feeding and induces a rhythmic sucking reflex^30^. Therefore, measuring the infants’ sucking pressure and sucking rhythm, as well as analyzing the results, would be possible to provide an objective assessment of the effects of exogenous oxytocin exposure on feeding behavior.

In this study, we hypothesized that intrapartum oxytocin exposure would weaken the infant’s ability to suck and negatively affect breastfeeding. This study aimed to determine the effects of intrapartum oxytocin administration on infants’ feeding behavior by measuring sucking pressure and rhythm, as well as to examine its effects on breastfeeding.

## Methods

### Participants

This study was conducted in a Comprehensive Maternity and Perinatal Care Center in Fukuoka, Japan. The hospital was not accredited by the Baby Friendly Hospital Initiative, and if the mothers needed to supplement their breast milk, formula was used. A total of 86 normal newborns born between May 2022 and April 2023, as well as their mothers, were recruited within 24–48 h of birth. To determine the effects of fetal exposure to exogenous oxytocin, participants were limited to singleton neonates born via vaginal delivery. Infants with congenital abnormalities, preterm infants, infants admitted to the neonatal intensive care unit (NICU) and infants with an Apgar score of less than 7 at 5 min were excluded. In addition, mothers with postpartum abnormalities, those with contraindications to breastfeeding, and those who wished to feed exclusively with formula prior to delivery were excluded. Mothers who met the research eligibility criteria were informed about the study and voluntary participation via an explanatory document, and their written informed consent to participate was obtained. The study protocol was approved by the Institutional Review Boards and Ethics Committees of Kyushu University Hospital and Medical Institutions (ethics approval number: 21131-01). The study was conducted in accordance with the WMA Declaration of Helsinki, the hospital’s and government guidelines and regulations.

### Data collection

#### Intrapartum oxytocin exposure status

Intrapartum oxytocin was administered for medical indications to augment or induce labor; a vial of OXYTOCIN injection 5IU (JAN, INN) was mixed with 500-ml 5% glucose infusion. Intravenous infusion was started at 2 mU/min and increased by 1 mU/min at intervals of at least 30 min until effective labor was achieved. The maximum dose was 20 mU/min. During the third stage of labor, a customary dose of 10-IU oxytocin was administered intravenously to all patients and increased as needed. Data on the amount and duration of oxytocin administration and reasons for oxytocin use were collected from medical records.

#### Variables related to breastfeeding and sucking ability

The following mother–infant variables were collected from the medical records and questionnaires. Maternal characteristics included maternal age, history of childbirth, postpartum depression, complications, and smoking history. Delivery information: duration of labor, blood loss at delivery, instrumental delivery, and use of analgesia during labor. Infant characteristics included birth weight, umbilical cord arterial blood pH, Apgar score, gestational age at birth, and sex.

#### Feeding for infants

The mothers were asked about their breastfeeding intentions on the first postpartum day. Data on the details of actual feeding methods for babies (exclusive breastfeeding/mixed breastfeeding and formula/exclusive formula) at 1 month postpartum were collected.

#### NNS

NNS was evaluated by having infants aged 24–48 h suckle a pacifier nipple for 5 min at least 2 h after the last feeding. Nipples were supplied by Pigeon Co., Ltd. (Tokyo, Japan) and are routinely used in hospitals to feed infants with formula. The hospital routinely added formula according to the mother's request until the mother's breast milk production increased after birth. Therefore, few infants had never suckled an artificial nipple at the time of NNS measurement at 24–48 h after delivery. However, it has been reported that introducing pacifiers to infants is associated with worse breastfeeding outcomes^31^. Therefore, to ensure that having the infant suck on the artificial nipple for 5 min for the study did not interfere with breastfeeding acquisition, we were careful to exclude infants who had never used an artificial nipple since birth. In addition, after fully explaining the study to the mothers, their consent to participate in the study was obtained. The nipple was modified to measure the intraoral negative pressure during sucking. A silicone tube with an inner diameter of 1 mm and a total length of 80 mm was attached to the hole punched in the center of the nipple. The other end of the tube was then connected to a micro-semiconductor pressure transducer (Keyence Co., Ltd., Osaka, Japan) and a data logger (Graphtec Co., Ltd., Yokohama, Japan).

Infants underwent measurements while lying on the neonatal bed of the hospital. The infant’s arousal level was recorded before the start of the NNS measurement using a 3-point scale (0 = asleep, 1 = quietly awake, 2 = crying). The infants were gently stroked on the side of their lips with a Silicone Rubber Nipple to elicit a rooting response. The nipple was gently placed in the mouth, and the sucking pressure and rhythm were measured for 5 min after the infants initiated the sucking cycle. Readiness to start sucking was also recorded using a 4-point scale (3 = sucking started within 1 min; 2 = within 1 to 3 min; 1 = after 3 min; 0 = sucking did not start).

**Figure 1:**
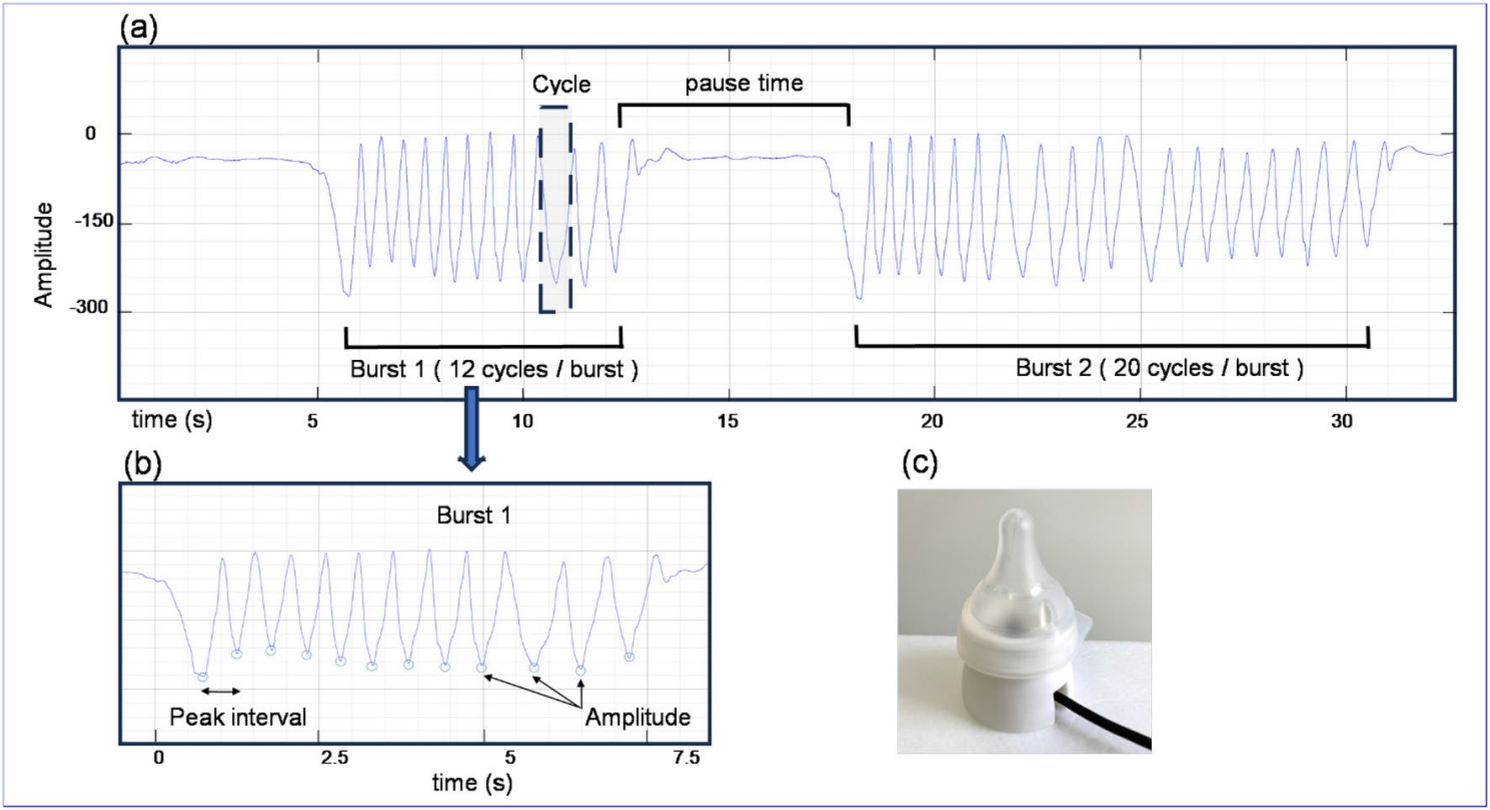
NNS measurements. *NNS* non-nutritive sucking. (**a**) Example NNS graph. Two bursts with a pause in between were observed in about 30 s. (**b**) Zoom of Burst 1. Amplitude means the peak value of intraoral negative pressure (mmHg). Peak interval means the length between the peak values of the intraoral negative pressure. (**c**) Left: modified nipple used for NNS measurements. Right: an infant undergoing NNS measurement.

Infants perform sucking movements in bursts (continuous sucking cycles) and pauses (periods of rest). NNS burst was defined as one burst containing two or more sucking amplitude cycles. A new next burst was defined as one in which the interval between sucking cycles was separated by more than 1000 ms with no obvious connection to the previous cycle. All NNS data were graphed using the software program AcqKnowledge 5.0 (BIOPAC Systems, Inc., Goleta, CA), and the mean values of the following temporal parameters and pressure variables were calculated (Fig. 1). To design a protocol for measuring and analyzing NNS, we referenced previous studies that investigated NNS^32–41^.

1. Total cycles (times): total number of sucking cycles per 5 min
2. Total bursts (times): total number of bursts per 5 min
3. Burst rate (%): percentage of total burst duration in 5 min
4. Amplitude (mmHg): peak value of intraoral negative pressure in the sucking cycle
5. Peak interval (s): interval between peak values of intraoral negative pressure
6. Pause time (s): interval between bursts
7. Burst duration (s): duration of each burst
8. Cycles/burst (time): number of cycles within a burst
9. Frequency (time): number of cycles per second within a burst

### Statistical analysis

Participants were divided into two groups: the With OXT group that was administered intrapartum oxytocin in the first and second trimesters of delivery, and the Without OXT group that was not administered intrapartum oxytocin in the first and second trimesters of delivery.

Descriptive statistics were calculated, and the differences in the distribution of maternal, infant, delivery, and breastfeeding characteristics between the two groups were determined. Multiple regression analysis was performed with NNS as the outcome variable and oxytocin exposure as the independent variable. As covariates, important variables reported in previous studies as influencing factors of infant feeding behavior were entered into the model. The presence of maternal complications was also included. Furthermore, we limited the variables after considering the number of cases and checking the VIF values between variables.

To assess the effect of oxytocin exposure on breastfeeding achievement through reduced sucking performance in infants, structural equation modeling (SEM) was used to estimate the path diagram, with NNS as a mediating factor in the causal relationship between oxytocin exposure and breastfeeding attainment. To evaluate the mediating effect of NNS, the significance of the indirect effect was tested using the bootstrap method (bootstrap sample size: 2000, confidence interval [CI]: 95%), and the standardized indirect effect and bootstrapping bias-corrected confidence intervals were calculated using SPSS Amos version 21.0 (IBM Corp., Armonk, NY, USA). The outcome variable was a binary variable of “exclusive breastfeeding/other,” which indicated breastfeeding achievement. Since many factors influence breastfeeding, univariate logistic regression analysis was conducted with “exclusive breastfeeding /other” as the dependent variable, and variables that showed *p* < 0.2 were extracted. In addition, representative variables that showed strong associations in previous studies were entered into the model diagram as control variables.

All *p*-values were two-sided, and statistical significance was considered at *p*-values < 0.05. Statistical analyses were performed using SPSS, version 27 (IBM Corp.) and SPSS Amos, version 21.0 (IBM Corp.).

## Results

### Participants

A total of 86 full-term infants and their postpartum mothers were eligible for this study. After the survey was completed, the following participants were excluded from the analysis (4 infants transferred to the NICU after the survey, 1 mother who had severe postpartum depression that made a longitudinal study impossible, 1 infant who did not produce sucking bursts during measurement, and 16 cases with a significant decrease in the NNS pressure waveform at baseline). Finally, 64 pairs of infants and their mothers were analyzed.

Normally, when infants perform sucking movements, the intraoral pressure values return to approximately the baseline values after the sucking cycle. In contrast, in cases where the baseline dropped, the negative intraoral pressure continued to increase for an unknown reason. These cases were excluded from the analysis because they could not be accurately measured. No background bias was observed in any of the excluded patients.

Of the 64 pairs analyzed, there were 29 from the With OXT group and 35 from the Without OXT group. In the With OXT group, 21 patients underwent planned labor induction and 8 underwent labor augmentation. The intrapartum oxytocin exposure times and doses are shown in Table 1. The objectives and indications for oxytocin administration are described in Supplementary Material 1.

**Table 1:** Basic characteristics of the with oxytocin and without oxytocin groups.

	Without OXT (n = 35)	With OXT (n = 29)	*p-*value
Maternal characteristics			
Maternal age (years)	32.20 ± 6.03	34.24 ± 3.15	0.14
History of childbirth			
Primipara	14 (40.0)	15 (51.7)	0.451
Multipara	21 (60.0)	14 (48.3)	
Postpartum depression			
EPDS score at discharge	3.40 ± 3.62	4.10 ± 3.77	0.279
EPDS score at 1 month checkup	2.63 ± 2.76	2.59 ± 2.98	0.794
Maternal complications			0.048*
Any	22 (62.9)	25 (86.2)	
None	13 (37.1)	4 (13.8)	
Smoking history, yes	5 (14.3)	1 (3.4)	0.312
Nipple shape problems for breastfeeding	4 (11.4)	7 (24.1)	0.203
Breast and nipple troubles at discharge	14 (63.6)	8 (27.6)	0.428
Feeding for infants			
Breastfeeding intentions (method of feeding that mother desired)			0.138
Exclusive breastfeeding	9 (25.7)	3 (10.4)	
Mixed breastfeeding and formula	26 (74.3)	26 (89.4)	
Actual feeding method (1 month postpartum)			0.033*
Exclusive breastfeeding	15(42.9)	5(17.2)	
Mixed breastfeeding and formula	18(51.4)	22(75.9)	
Exclusive formula	2 (5.7)	2 (6.9)	
Delivery information			
Duration of labor (minutes)	349.46 ± 236.81	376.79 ± 273.28	0.819
Blood loss at delivery (ml)	522.75 ± 279.71	515.10 ± 264.52	0.356
Instrumental delivery	2 (5.7)	6 (20.7)	0.127
Use of epidural analgesia in labor	0 (0.0)	4 (13.8)	0.037*
Intrapartum oxytocin use			
Oxytocin administration dose (IU)	–	3.45 ± 5.28	
Oxytocin administration time (minutes)	–	354.38 ± 428.53	
Infant characteristics			
Birth weight (g)	3152.66 ± 407.90	3035.97 ± 377.32	0.272
Umbilical cord arterial blood pH value	7.309 ± 0.051	7.316 ± 0.092	0.691
APGAR score			
1 min	8.09 ± 0.387	8.12 ± 0.485	0.847
5 min	9.02 ± 0.218	9.09 ± 0.412	0.094
Gestational age at birth (weeks)	39.3 ± 0.9	38.6 ± 1.0	0.077
Infant sex			0.451
Male	14 (40.0)	15 (51.7)	
Female	21 (60.0)	14 (48.3)	
Time since birth	34.80 ± 7.39	38.93 ± 8.42	0.052

The data shown are mean ± SD or number of people (%).

The *p*-values were estimated from the Mann–Whitney* U* test or chi-squared test; *p < 0.05, **p < 0.01, ***p < 0.001.

*SD* standard deviation, *EPDS* Edinburgh postnatal depression scale.

Table 1 reports maternal and infant characteristics, feeding practices, and delivery information stratified by the With and Without OXT group. The percentage of mothers with complications was significantly higher in the With OXT group than in the Without OXT group (*p* = 0.048). In this study, maternal complications were defined as follows. Maternal complications: Participants with existing medical conditions under observation or pregnancy complications^42,43^. No maternal complications: participants not applicable to maternal complications.

Details of maternal complications in this study are given in Table 2.

**Table 2:** Details of maternal complications.

	Without OXT		With OXT		n = 64
	N	%	n	%	*P-*value
Maternal complications					
Hypertensive disorders of pregnancy	0	0.0	3	10.3	0.088
Gestational or maternal diabetes	5	14.3	9	31.0	0.135
Threatened preterm labor	1	2.9	2	6.9	0.586
Cardiac disease	1	2.9	4	13.8	0.167
Kidney disease	1	2.9	2	6.9	0.571
Thyroid disease	2	5.7	2	6.9	1
Epilepsy	1	2.9	1	3.4	1
Other complications^a^	9	25.7	2	6.9	0.093
No complications	13	37.1	4	13.8	0.048*

The *p*-values were estimated from the Chi-squared test; *p < 0.05.

*OXT* oxytocin.

^a^Other complications: gathered complications of only one person. The complications included low lying placenta, keratitis, history of venous thromboembolism, excision of vulvar condyloma, scoliosis, citrin deficiency, history of hepatitis B, positive anti-SSA antibody, cerebral hemangioma, and history of intestinal obstruction.

The number of cases per complication was small. Only participants with well-controlled maternal complications were included in the study, there were no patients with severe conditions that interfered with breastfeeding. In this study, although there were differences in the distribution of maternal complications between the two groups, we carefully ensured that the rates of NNS and breastfeeding achievement were similar between the groups with and without main complications was similar and adjusted for these complications in the multiple regression analysis of NNS patterns. The results are shown in Supplementary Material 2–9.

Supplementary Material 10 reports the information on the infants at the time of the NNS survey. There were no significant differences in arousal level and time required to start sucking between the With and Without OXT groups.

Table 1 also reports the data on breastfeeding practices at 1 month postpartum, grouped into “Exclusive breastfeeding”, “Mixed breastfeeding and formula”, and “Exclusive formula”. The rate of the exclusively breastfeeding group at 1 month postpartum was significantly higher in the Without OXT group than in the With OXT group (*p* = 0.033). Details of the feeding method are shown in Supplementary Material 11.

### Impact of intrapartum oxytocin exposure on NNS

Table 3 reports the results of the comparison of NNS measurements between the With and Without OXT groups. The With OXT group had significantly fewer total bursts (*p* = 0.041), significantly longer pause times (*p* = 0.044), and a significantly larger coefficient of variation (CV%) for pause times than the Without OXT group (*p* = 0.040).

**Table 3:** NNS measurements of the with oxytocin and without oxytocin groups.

	Without OXT (n = 35)				With OXT (n = 29)				*p-*value
	Mean	SD	Median	IQR	Mean	SD	Median	IQR	
Total cycles	209.94	93.82	219.00	173.00	182.03	92.31	181.00	151.00	0.252
**Total bursts**	**17.74**	**6.40**	**18.00**	**7.00**	**14.38**	**5.94**	**14.00**	**7.50**	0.041*
Burst rate	40.17	21.01	42.94	36.26	36.06	19.51	31.63	25.92	0.557
Amplitude	−189.23	35.26	−184.70	42.45	−193.61	37.62	−192.82	57.10	0.415
CV%^a^	19.08	7.28	19.08	11.40	19.80	6.73	18.17	7.40	0.531
Peak interval	0.62	0.08	0.60	0.14	0.64	0.08	0.63	0.10	0.171
CV%^a^	19.40	3.33	19.60	4.01	19.44	3.54	19.44	4.29	0.887
**Pause time**	**11.01**	**6.40**	**8.52**	**5.07**	**14.88**	**9.79**	**12.27**	**6.64**	0.044*
**CV%**^a^	**78.28**	**49.68**	**69.16**	**75.96**	**104.47**	**54.99**	**84.97**	**109.49**	0.040*
Burst duration	7.94	7.37	6.67	5.97	7.79	4.04	7.79	7.62	0.446
CV%^a^	70.39	27.04	66.52	43.48	69.24	28.47	67.49	34.22	0.772
Cycles/burst	13.24	9.53	11.35	9.06	12.94	5.73	12.86	8.90	0.496
CV%^a^	61.35	26.42	58.84	33.91	59.67	26.41	55.10	28.20	0.681
Frequency	2.04	0.36	1.98	0.49	1.94	0.37	1.88	0.50	0.243
CV%^a^	19.28	10.59	17.28	12.05	17.83	9.43	17.56	14.92	0.731

The *p*-values were estimated from the Mann–Whitney U test; *p < 0.05, **p < 0.01, ***p < 0.001.

*SD* standard deviation, *CV* coefficient of variation.

^a^CV% = (SD/mean × 100).

Significant values are in bold.

Table 4 reports the results of a multiple linear regression analysis of the impact of intrapartum oxytocin exposure on NNS. In the model adjusted for covariates, oxytocin exposure was significantly associated with fewer total bursts (95% CI, -7.02 to -0.22), longer pause time (95% CI, 1.33 to 10.21), and bigger CV% of pause time (95% CI, 3.63 to 63.92).

**Table 4:** Impact of intrapartum oxytocin exposure on NNS.

	Simple linear regression model^a^					Multiple linear regression model^b^				
Outcomes	B	SE	Β	*p*-value	95% CI	B	SE	Β	*p*-value	95% CI
Total bursts	−3.66	1.53	v0.29	0.021*	[−6.68 to −0.57]	−3.62	1.70	−0.29	0.037*	[−7.02 to −0.22]
Pause time	3.87	2.04	0.24	0.062	[−0.20 to 7.94]	5.77	2.22	0.35	0.012*	[1.33 to 10.21]
Pause time CV%	26.20	13.09	0.25	0.050*	[0.02 to 52.37]	33.77	15.05	0.32	0.029*	[3.63 to 63.92]

Multiple regression analysis with oxytocin exposure as the independent variable for NNS outcomes with significant differences between the two groups with and without oxytocin.

*B* partial regression coefficient, *SE* standard error, *β* standardized partial regression coefficient, *CI* confidence interval, *NNS* non-nutritive sucking.

^a^Only oxytocin exposure was entered as an independent variable.

^b^Infant's birth weight, infant's gestational age at birth, infant's sex, maternal age, duration of labor, and presence of maternal complications were entered as covariates.

### Relationship between NNS-mediated intrapartum oxytocin exposure and breastfeeding

**Figure 2:**
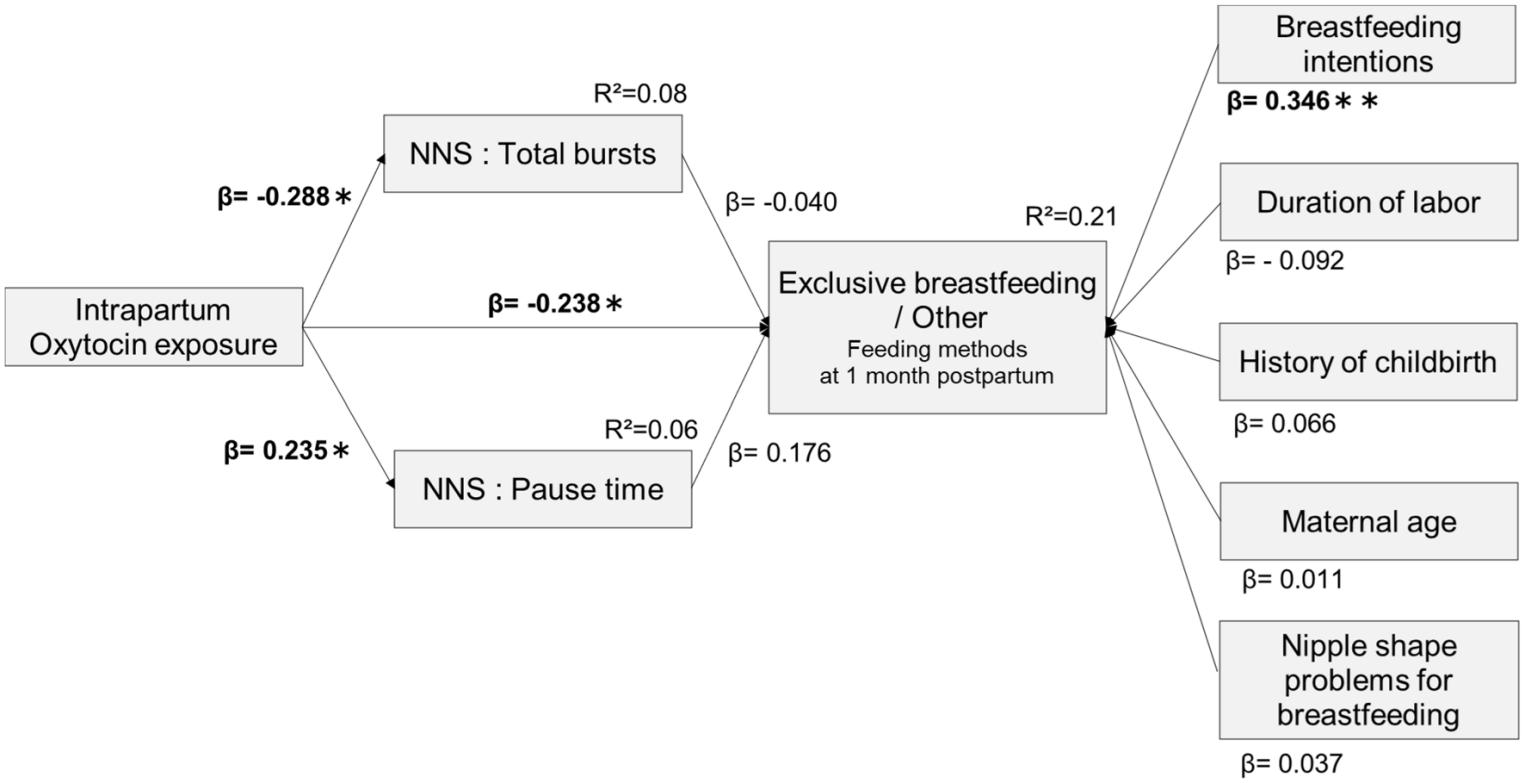
Path diagram—impact of oxytocin exposure and NNS on feeding methods at 1 month postpartum. *β* standardized partial regression coefficient, *EPDS* Edinburgh postnatal depression scale. Exclusive breastfeeding: 1/other: 0

Path models with the NNS variable as a mediator of the causal relationship between oxytocin exposure and feeding methods (exclusive breastfeeding/other) at 1 month postpartum are shown in Fig. 2. The standard errors and *p*-values for the direct effect estimates are presented in Supplementary Material 12. Factors influencing breastfeeding in addition to NNS were narrowed down based on variables from previous studies and the results of single regression analysis and entered into the model diagram (Supplementary Material 13). The standardized partial regression coefficients are the values of the direct effect coefficients. Significant negative effects (β = −0.288, *p* = 0.017) from oxytocin exposure to total bursts and significant positive effects (β = 0.235, *p* = 0.050) on pause-time length were observed. There was also a significant direct effect from intrapartum oxytocin exposure to the feeding methods at 1 month postpartum (β = −0.238, *p* = 0.047). However, there was no significant direct effect of total bursts on the feeding methods at 1 month postpartum (β = −0.040, *p* = 0.734). Pause time to the feeding methods at 1 month postpartum (β = 0.176, *p* = 0.127) also indicated no significant direct effect. Breastfeeding intentions as a factor influencing breastfeeding were strongly associated with 1-month postpartum feeding methods (β = 0.346, *p* = 0.001).

Table 5 presents the estimates of the indirect effects. There was no significant indirect effect of oxytocin exposure through the NNS burst on the breastfeeding method at 1 month postpartum (bootstrap bias-corrected 95% CI, −0.083 to 0.109). There was also no significant indirect effect of the NNS pause time (bootstrap bias-corrected 95% CI, −0.018 to 0.176).

**Table 5:** Multiple mediation model: indirect effects of oxytocin on 1-month postpartum feeding methods through NNS.

Relationship	Indirect effect (products of coefficients)				Bootstrapping bias-corrected 95% CI
	B	SE	β	*p*-value	95% CI
Oxytocin → total bursts → exclusive breastfeeding/other	0.01	0.05	0.01	0.726	[-0.08–0.11]
Oxytocin → pause time → exclusive breastfeeding/other	0.04	0.05	0.04	0.209	[-0.02–0.18]

Bootstrap sample size = 2000.

*B* partial regression coefficient, *SE* standard error, *β* standardized partial regression coefficient, *CI* confidence interval, *NNS* non-nutritive sucking.

## Discussion

The components of infant feeding are the state of arousal, tactile and olfactory functional systems to find the mother’s nipple, routing reflex, rhythmic sucking, and swallowing^30^. This study objectively evaluated infant sucking ability, a part of the feeding behavior, using NNS measurements. Infants exposed to oxytocin in utero exhibited significantly fewer total bursts, longer pause times, and significantly larger variations in pause times than those not exposed to oxytocin. This is a new finding, as no previous studies have reported that oxytocin exposure affects NNS.

This study examined the effects of fetal exposure to NNS. Previous studies have investigated the association between NNS and fetal exposure to substances other than oxytocin. Zimmerman et al.^35^ examined the association between phthalate exposure during pregnancy and NNS. They reported a lower sucking frequency and higher amplitude in the exposed group of infants. It has been suggested that the amplitude may have increased to compensate for the slower sucking frequency. Additionally, higher maternal prenatal stress values were found to be associated with fewer infant NNS bursts/minute and longer burst duration^40^. Although the mechanism by which the in-utero environment negatively affects the sucking ability of the child is not yet clear, these previous studies have described the possibility that the in-utero environment affects fetal neurodevelopment and reduces the generation of sucking patterns. Similar to reports of fetal cardiopulmonary function changes and decreases in fetal movement in the presence of stress in the prenatal environment^44^, NNS vitality may decrease as a self-soothing mechanism.Additionally, the NNS of infants who developed sequelae later has been shown to have fewer sucking bursts per minute, slower sucking frequency, and greater variability in frequency and amplitude^38^. Compared to these results, the sucking ability of the With OXT group, which showed fewer bursts and longer, more variable pause times, was more similar to that of the sick infants and can be considered a weak sucking pattern.

The mechanisms by which oxytocin exposure influences the NNS have been discussed. Endogenous oxytocin acts on motor neurons, induces sucking, and plays an important role in feeding^45^. The source of endogenous oxytocin in the fetus is produced by the fetus itself, while the maternal oxytocin passes through the immature blood–brain barrier of the fetus^46^. There are two barriers to oxytocin influx into the neonatal brain: the placental barrier and the cerebral blood barrier, which contains oxytocinase, an enzyme involved in the breakdown of oxytocin^5,46^. Although these barriers may inhibit the passage of peptides such as oxytocin, they are not fully mature and may be highly permeable during the fetal period^21^. Phaneuf et al.^47^ observed that prolonged oxytocin exposure decreased oxytocin receptor utilization by decreasing OT receptor messenger RNA levels and caused desensitization. Fetal oxytocin exposure may cause downregulation of the innate oxytocin rhythm that acts on fetal sucking rhythm expression, resulting in a weakening of the sucking pattern. In addition, a meta-analysis of animal studies reported an association between exogenous oxytocin administration, suppression of food and water intake, and reduced mealtimes^48^. The mechanism is not fully understood; however, the few sucking expressions observed in this study may also be explained by an appetite-decreasing effect. However, there are mixed views on whether exogenous oxytocin reaches the fetus at clinical doses. A recent systematic review^49^ of studies measuring maternal oxytocin levels, as well as cord arterial and venous oxytocin levels, found that oxytocin levels in cord blood do not differ with or without synthetic oxytocin administration during delivery and may not be transmitted from the mother to the fetus. It has been reported that five of the studies under review were from small samples from the 1970s and 1980s, and one of the studies reported lower umbilical artery oxytocin levels (24.6 pg/mL) in newborns whose mothers received synthetic oxytocin than in controls (116 pg/mL). However, this review is an important report. Other narrative review articles^12^ included studies that examined oxytocin levels and neonatal behavior in animals after perinatal oxytocin manipulation and the possibility of placental transport of oxytocin. This review suggested that exposure to exogenous oxytocin may affect various of basic neurological systems and behaviors, including protection against fetal hypoxia, initiating and regulating neonatal feeding, and early social behavior. However, the final conclusion was that the knowledge of the conditions under which peripheral oxytocin signaling reaches the fetus is incomplete. Thus, conflicting evidence is mixed, and no clear consensus has been reached. Further research is needed to obtain evidence that clinical doses of exogenous oxytocin reach the fetus and disrupt the endogenous oxytocin rhythm.

In this study, to examine a hypothetical model in which intrapartum oxytocin exposure affects breastfeeding achievement mediated by NNS, the effect was estimated using SEM. The results suggested that the influence of the NNS pattern attenuated by oxytocin exposure was not strong enough to affect breastfeeding achievement. Breastfeeding is a mother–infant interaction, and the mother's endogenous oxytocin responds to effective sucking by the infant. Repeated and effective breastfeeding stimulates breast milk secretion, thereby facilitating breastfeeding. The sucking pattern of the With OXT group, which had fewer sucking bursts and longer pauses than the Without OXT group, predicted a weaker approach to maternal endogenous oxytocin and weaker breastfeeding achievement; however, the results of this study did not show this effect. The NNS patterns of infants were measured at a one time, 24–48 h after birth. Assuming that the negative effect of oxytocin on sucking was transient, it is possible that the later sucking ability did not differ between the two groups and did not affect breastfeeding achievement. Additionally, we did not observe in this study whether infants reproduce the same sucking patterns as the characteristics shown in the NNS when feeding from the breast or bottle. Further and longitudinal studies are required to confirm this hypothesis.

Some studies reported a negative association between intrapartum oxytocin exposure and breastfeeding practices^15–19^, whereas others reported no such association^20,21^. In this study, we found no indirect effects on breastfeeding via attenuated NNS; however, we did find a direct and negative effect of oxytocin exposure on breastfeeding. This finding suggests that factors other than the infant's sucking ability may affect breastfeeding.

Previous studies have discussed factors that negatively affect breastfeeding due to intrapartum oxytocin exposure, including interruption of breastfeeding initiation, decreased ejaculatory reflex due to downregulation of the endogenous oxytocin system^5^, and inhibition of lactation due to an enhanced stress response^50^. It has also been reported that mothers who receive intrapartum oxytocin secrete lower levels of endogenous oxytocin during postpartum breastfeeding^51^. Further accumulation of evidence is expected.

This is the first study to evaluate the effect of intrapartum oxytocin on sucking movements using objective parameters. It is clear that a strategy of inducing labor at or beyond term is associated with fewer perinatal deaths and fewer cesarean sections^52,53^, and it is imperative in healthcare practice to induce and augment labor according to medical indications. However, breastfeeding should be supported while considering that infants born to mothers exposed to intrapartum oxytocin may have an attenuated feeding ability, at least until the second day after birth.

### Study strength and limitation

The strength and originality of this study is that the impact of intrapartum oxytocin exposure on infant feeding behavior was objectively evaluated using a sucking measurement approach. The study was conducted in a university hospital providing advanced medical care as the study field. Although the cohort included mothers with maternal complications, only patients who met the eligibility criteria and whose maternal complications were well-controlled were included in the analysis. In addition, we ensured that the rates of NNS and breastfeeding achievement were similar between the groups with and without major complications and adjusted for these complications in the multiple regression analysis of NNS characteristics. However, the possibility that maternal complications influenced the results cannot be ruled out. A limitation of this study is that the backgrounds of the two study groups were not controlled completely, limiting the generalizability of the results. Further studies are needed to accumulate data on participants with no risk in the future. In addition, due to the small sample size, entering all possible predicted influencing factors into the analytical model was impossible. This is an observational study, and a limitation of this study is that we cannot rule out the possibility that unmeasured confounding factors may have influenced the results.

## Conclusions

This study investigated the effects of intrapartum oxytocin exposure on infant sucking ability (NNS) during the first 24–48 h after birth and breastfeeding at one month postpartum. In adjusted multiple regression models, intrapartum oxytocin exposure was significantly associated with fewer total NNS bursts, longer pause times, and greater pause-time variability. This suggesting that intrapartum oxytocin exposure may have a negative effect on infant sucking ability is a novel finding. The effects estimated using SEM showed a significant, negative, and direct effect of intrapartum oxytocin exposure on the practice of exclusive breastfeeding 1 month postpartum. However, no indirect effects of NNS were found, rejecting the hypothesis that intrapartum oxytocin exposure attenuates infants' sucking ability and thus negatively affects breastfeeding. It is necessary to consider that infants born to mothers who receive intrapartum oxytocin may have attenuated sucking ability for at least the first 48 h after birth, and breastfeeding support should be provided.

### Supplementary Information

Supplementary Information.

## Data Availability

Data supporting the findings of this study are available from the corresponding author upon request.
