# Supplementary material for: Impact of intrapartum oxytocin administration on neonatal sucking behavior and breastfeeding
Source: Sci Rep. 2024 Mar 11;14:5859. doi: 10.1038/s41598-024-56635-9 (PMC10928222; doi:10.1038/s41598-024-56635-9)
Supplement: Supplementary file 1 — Supplementary Information. [file 41598_2024_56635_MOESM1_ESM.pdf]

Objective	Indications	n (%)
Augmentation of labor	Delayed labor	8 (100)
	Overall	8 (100)
Induction of labor	Maternal gestational diabetes, diabetes	5 (23.8)
	Pre-labor rupture of membranes	4 (19.0)
	Maternal cardiac disease	4 (19.0)
	Maternal hypertensive disorders of pregnancy	2 (9.5)
	Family preferences	2 (9.5)
	Past the expected date of delivery	1 (4.8)
	Maternal kidney disease	1 (4.8)
	Maternal epilepsy	1 (4.8)
	Risk of rapid delivery progress	1 (4.8)
	Overall	21 (100)

Supplementary Material 1: Objectives and indications for oxytocin administration

n=64

Major maternal complications	YES<br>Exclusive breastfeeding /others†	NO<br>Exclusive breastfeeding /others†	*P*-value
Hypertensive disorders of pregnancy	0 (0.0) / 3 (100.0)	20 (32.8) / 41 (67.2)	0.318
Gestational or maternal diabetes	4 (28.6) / 10 (71.4)	16 (32.0) / 34 (68.0)	0.542
Threatened preterm labor	1 (33.3) / 2 (66.7)	19 (31.1) / 42 (68.9)	0.682
Cardiac disease	1 (20.0) / 4 (80.0)	19 (32.2) / 40 (67.8)	0.499
Kidney disease	1 (33.3) / 2 (66.7)	19 (31.1) / 42 (68.9)	0.682
Thyroid disease	1 (25.0) / 3 (75.0)	19 (31.7) / 41 (68.3)	0.631
Epilepsy	1 (50.0) / 1 (50.0)	19 (30.6) / 43 (69.4)	0.531

Supplementary Material 2. Percentage of mothers exclusively breastfeeding at 1 month postpartum in the groups with and without complications.

†others mean mixed breastfeeding and formula and exclusive formula.

Data are numbers and percentages.

P-values were calculated using the chi-squared test.

n=64

Hypertensive disorders of pregnancy	**Yes (n=3)** Mean	SD	**No (n=61)** Mean	SD	*p*-value
Total cycles	186.33	(52.00)	202.75	(91.84)	0.444
**Total bursts**	17.00	(3.61)	16.64	(6.07)	0.130
Burst rate	37.89	(12.04)	39.31	(20.14)	0.780
Amplitude	-182.15	(34.49)	-188.22	(36.77)	0.763
CV%†	-18.53	(3.81)	-19.43	(7.13)	0.812
Peak interval	0.63	(0.09)	0.63	(0.08)	0.812
CV%†	19.81	(6.62)	19.40	(3.27)	0.787
**Pause time**	18.02	(8.61)	12.51	(8.24)	0.109
**CV%†**	149.64	(39.89)	87.22	(52.47)	0.055
Burst duration	6.91	(2.35)	7.92	(6.18)	0.886
CV%†	83.13	(38.18)	69.22	(27.11)	0.418
Cycles/burst	11.67	(2.36)	13.18	(8.15)	0.911
CV%†	71.95	(43.99)	60.03	(25.50)	0.515
Frequency	2.02	(0.20)	1.99	(0.37)	0.715
CV%†	25.11	(6.29)	18.30	(10.10)	0.131

Supplementary Material 3. Non-nutritive sucking characteristics in infants born to mothers with and without hypertensive disorders of pregnancy.

P-values were calculated using the Mann–Whitney U test.

†CV%=(SD/mean×100).

Abbreviations: SD, standard deviation; CV, coefficient of variation.

n=64

Gestational or maternal diabetes	**Yes (n=14)** Mean	SD	**No (n=50)** Mean	SD	*p*-value
Total cycles	217.21	(92.27)	191.72	(93.94)	0.389
**Total bursts**	17.07	(4.80)	15.94	(6.68)	0.505
Burst rate	40.53	(18.23)	37.69	(20.96)	0.570
Amplitude	-193.14	(39.12)	-186.47	(35.92)	0.733
CV%†	-20.46	(7.27)	-19.09	(6.96)	0.526
Peak interval	0.61	(0.08)	0.63	(0.08)	0.291
CV%†	19.06	(2.92)	19.51	(3.55)	0.649
**Pause time**	11.18	(6.17)	13.21	(8.77)	0.372
**CV%†**	80.13	(37.82)	92.95	(56.98)	0.909
Burst duration	7.31	(3.59)	8.03	(6.59)	0.974
CV%†	66.33	(19.36)	70.86	(29.44)	0.845
Cycles/burst	12.77	(5.11)	13.20	(8.65)	0.685
CV%†	57.74	(18.86)	61.38	(28.04)	0.909
Frequency	2.01	(0.34)	1.98	(0.37)	0.697
CV%†	16.77	(7.51)	19.14	(10.63)	0.559

Supplementary Material 4. Non-nutritive sucking characteristics in infants born to mothers with and without gestational or maternal diabetes.
P-values were calculated using the Mann–Whitney U test.
†CV%=(SD/mean×100).

Abbreviations: SD, standard deviation; CV, coefficient of variation.

n=64

Threatened preterm labor	**Yes (n=3)**		**No (n=61)**		
	Mean	SD	Mean	SD	*p*-value
Total cycles	231.33	(82.95)	195.62	(94.24)	0.587
**Total bursts**	20.67	(4.04)	15.97	(6.33)	0.143
Burst rate	44.69	(19.46)	37.99	(20.43)	0.566
Amplitude	-188.80	(25.13)	-187.89	(37.04)	0.929
CV%†	-13.68	(4.44)	-19.67	(7.00)	0.088
Peak interval	0.63	(0.06)	0.63	(0.08)	0.788
CV%†	19.52	(4.44)	19.41	(3.39)	0.976
**Pause time**	7.92	(1.22)	13.00	(8.40)	0.182
**CV%†**	69.36	(52.68)	91.17	(53.63)	0.587
Burst duration	7.00	(3.95)	7.91	(6.15)	0.905
CV%†	50.21	(4.25)	70.84	(27.79)	0.204
Cycles/burst	11.91	(5.66)	13.16	(8.10)	0.881
CV%†	43.16	(7.39)	61.45	(26.53)	0.193
Frequency	1.87	(0.27)	2.00	(0.37)	0.674
CV%†	11.56	(3.33)	18.97	(10.13)	0.161

Supplementary Material 5. Non-nutritive sucking characteristics in infants born to mothers with and without a history of threatened preterm labor.
P-values were calculated using the Mann–Whitney U test.
†CV%=(SD/mean×100).

Abbreviations: SD, standard deviation; CV, coefficient of variation.

n=64

Cardiac diseases	**Yes (n=5)**		**No (n=59)**		
	Mean	SD	Mean	SD	*p*-value
Total cycles	228.80	(54.98)	194.63	(95.84)	0.416
**Total bursts**	17.20	(5.93)	16.10	(6.37)	0.539
Burst rate	44.08	(11.32)	37.82	(20.86)	0.461
Amplitude	179.92	(36.64)	188.61	(36.64)	0.608
CV%†	21.22	(8.23)	19.23	(6.94)	0.644
Peak interval	0.62	(0.05)	0.63	(0.08)	0.950
CV%†	21.97	(2.91)	19.20	(3.37)	0.056
**Pause time**	13.06	(8.80)	12.74	(8.31)	0.990
**CV%†**	92.85	(63.72)	89.92	(53.04)	0.950
Burst duration	8.31	(3.03)	7.83	(6.25)	0.445
CV%†	76.02	(29.21)	69.35	(27.53)	0.557
Cycles/burst	14.10	(3.71)	13.02	(8.25)	0.388
CV%†	68.69	(28.79)	59.90	(26.14)	0.431
Frequency	1.90	(0.24)	2.00	(0.37)	0.774
CV%†	21.35	(11.75)	18.39	(9.95)	0.476

Supplementary Material 6. Non-nutritive sucking characteristics in infants born to mothers with and without cardiac diseases.

P-values were calculated using the Mann–Whitney U test.

†CV%=(SD/mean×100).

Abbreviations: SD, standard deviation; CV, coefficient of variation.

n=64

Kidney diseases	Yes (n=3)		No (n=61)		
	Mean	SD	Mean	SD	*p*-value
Total cycles	184.33	(64.29)	197.93	(94.99)	0.788
**Total bursts**	12.33	(1.53)	16.38	(6.39)	0.204
Burst rate	40.74	(10.47)	38.19	(20.70)	0.858
Amplitude	-214.82	(21.52)	-186.61	(36.61)	0.143
CV%†	-15.59	(1.86)	-19.57	(7.10)	0.446
Peak interval	0.74	(0.12)	0.62	(0.08)	0.059
CV%†	16.03	(0.62)	19.58	(3.40)	0.046*
**Pause time**	15.13	(4.52)	12.65	(8.42)	0.182
**CV%†**	73.72	(45.94)	90.96	(53.91)	0.719
Burst duration	9.86	(2.10)	7.77	(6.17)	0.182
CV%†	67.76	(34.39)	69.97	(27.45)	0.858
Cycles/burst	14.76	(4.00)	13.02	(8.13)	0.341
CV%†	59.99	(32.56)	60.62	(26.20)	0.976
Frequency	1.78	(0.56)	2.00	(0.35)	0.295
CV%†	27.32	(25.29)	18.19	(8.96)	0.834

Supplementary Material 7. Non-nutritive sucking characteristics in infants born to mothers with and without kidney diseases.

P-values were calculated using the Mann–Whitney U test. *$p<0.05$

†CV%=(SD/mean×100).

Abbreviations: SD, standard deviation; CV, coefficient of variation.

n=64

Thyroid diseases	Yes (n=4)		No (n=60)		
	Mean	SD	Mean	SD	*p*-value
Total cycles	161.00	(52.45)	199.72	(95.35)	0.397
**Total bursts**	16.25	(3.40)	16.18	(6.47)	0.989
Burst rate	31.44	(15.32)	38.77	(20.60)	0.565
Amplitude	-204.68	(14.06)	-186.82	(37.24)	0.298
CV%†	-19.84	(5.69)	-19.35	(7.11)	0.698
Peak interval	0.63	(0.08)	0.63	(0.08)	0.758
CV%†	21.07	(1.39)	19.31	(3.47)	0.120
**Pause time**	11.99	(1.38)	12.82	(8.54)	0.444
**CV%†**	117.79	(43.08)	88.31	(53.78)	0.227
Burst duration	5.96	(4.09)	8.00	(6.16)	0.428
CV%†	104.35	(46.81)	67.57	(24.68)	0.100
Cycles/burst	9.95	(4.89)	13.32	(8.12)	0.367
CV%†	88.25	(38.08)	58.74	(24.59)	0.100
Frequency	2.03	(0.42)	1.99	(0.36)	0.799
CV%†	17.59	(3.00)	18.69	(10.34)	0.904

Supplementary Material 8. Non-nutritive sucking characteristics in infants born to mothers with and without thyroid diseases.

P-values were calculated using the Mann–Whitney U test.

†CV%=(SD/mean×100).

Abbreviations: SD, standard deviation; CV, coefficient of variation.

					n=64
Epilepsy	**Yes (n=2)**		**No (n=62)**		
	Mean	SD	Mean	SD	*p*-value
Total cycles	100.00	(53.74)	200.44	(93.02)	0.168
**Total bursts**	13.50	(6.36)	16.27	(6.33)	0.595
Burst rate	15.31	(7.88)	39.05	(20.16)	0.089
Amplitude	-167.77	(15.83)	-188.58	(36.78)	0.339
CV%†	-29.46	(0.78)	-19.06	(6.86)	0.036*
Peak interval	0.54	(0.02)	0.63	(0.08)	0.049*
CV%†	20.84	(0.82)	19.37	(3.45)	0.339
**Pause time**	17.68	(5.02)	12.61	(8.33)	0.155
**CV%†**	151.46	(46.67)	88.17	(52.72)	0.143
Burst duration	3.36	(0.16)	8.01	(6.09)	0.131
CV%†	60.45	(28.35)	70.17	(27.64)	0.750
Cycles/burst	7.28	(0.55)	13.29	(8.03)	0.223
CV%†	46.43	(29.45)	61.05	(26.25)	0.458
Frequency	2.44	(0.27)	1.98	(0.36)	0.089
CV%†	24.43	(10.11)	18.43	(10.05)	0.377

Supplementary Material 9: Non-nutritive sucking characteristics in infants born to mothers with and without epilepsy.

P-values were calculated using the Mann–Whitney U test; *p<0.05.

†CV%=(SD/mean×100).

Abbreviations: SD, standard deviation; CV, coefficient of variation.

n=64

	Without OXT (n=35)	With OXT (n=29)	*p*-value
Infant's arousal level			0.251
Asleep	23 (65.7)	19 (65.5)	
Quietly awake	3 (8.6)	6 (20.7)	
Crying	9 (25.7)	4 (13.8)	
Time required to start sucking			0.586
Within 1 minute	21 (60.0)	15 (51.7)	
Within 1–3 minutes	4 (11.4)	6 (20.7)	
After 3 minutes	10 (28.6)	8 (27.6)	

Supplementary Material 10: Information on infants at the time of NNS measurements
The data shown are mean±SD or number of people (%).
The *p*-values were estimated from the chi-squared test.
Abbreviations: OXT, oxytocin.

n=64

		**Without Oxytocin n=35**		**With Oxytocin n=29**		*p*-value
Details of feeding methods		Mean	SD	Mean	SD	
72 hours postpartum (during hospitalization)	Total number of supplemental formula use from birth (times)	12.97	(6.37)	15.59	(6.54)	0.101
	Total amount of supplemental formula from birth (ml)	216.14	(131.81)	247.59	(116.62)	0.068
	Total number of breastfeeding from birth (times)	22.00	(5.35)	20.41	(5.97)	0.417
1 month postpartum	Number of supplemental formula use/ day(times)	**2.51**	**(2.99)**	**4.45**	**(2.98)**	**0.018***
	Amount of supplemental formula /day (ml)	**207.71**	**(267.14)**	**335.34**	**(268.16)**	**0.031***
	Number of breastfeeding/day (times)	8.18	(3.05)	7.52	(3.03)	0.375

Supplementary Material 11: Details of feeding methods during hospitalization and one month postpartum.
The *p*-values were estimated from the Mann-Whitney U test; *$p<0.05$.
Abbreviations: SD, standard deviation.

	β	SE	*p*-value
OXT→ Total bursts	-0.29	1.52	0.017*
OXT→ Pause time	0.24	2.02	0.05*
OXT→ Exclusive breastfeeding/Other	-0.24	0.11	0.047*
Total bursts → Exclusive breastfeeding/Other	-0.04	0.01	0.734
Pause time → Exclusive breastfeeding/Other	0.18	0.01	0.127
Breastfeeding intentions → Exclusive breastfeeding/Other	0.35	0.09	0.001**
Duration of labor → Exclusive breastfeeding/Other	-0.09	0.00	0.413
History of childbirth → Exclusive breastfeeding/Other	0.07	0.10	0.554
Maternal age → Exclusive breastfeeding/Other	0.01	0.01	0.924
Nipple shape problems for breastfeeding → Exclusive breastfeeding/Other	0.04	0.14	0.742

Supplementary Material 12: Estimated direct effects in the path diagram.
*$p<0.05$ **$p<0.01$ ***$p<0.001$
Abbreviations: β, standardized partial regression coefficient; SE, standard error; OXT, oxytocin.
Exclusive breastfeeding:1/Other:0

Predictor Variables	Exclusive breastfeeding:1/Other:0			
	B	Exp(B)	*p*-value	95% CI
**Oxytocin exposure**	-1.281	0.278	**0.032***	[0.086–0.898]
**Maternal factor**				
Age	-0.056	0.946	0.303	[0.851–1.051]
Smoking history	0.427	1.533	0.547	[0.382–6.155]
EPDS score at discharge	-0.861	1.020	0.789	[0.885–1.175]
History of childbirth	-1.145	1.857	0.267	[0.623–5.537]
Maternal complications				
Hypertensive disorders of pregnancy	-20.485	0.000	0.999	–
Gestational or maternal diabetes	-0.163	0.850	0.807	[0.231–3.128]
Epilepsy	0.817	2.263	0.571	[0.134–38.117]
Cardiac disease	-0.642	0.526	0.577	[0.055–5.035]
Kidney disease	0.100	1.105	0.936	[0.094–12.948]
Thyroid disease	-0.329	0.719	0.781	[0.070–7.375]
Threatened preterm labor	0.100	1.105	0.936	[0.094–12.948]
Other complications	0.279	1.321	0.688	[0.339–5.155]
No complications	0.953	2.593	0.206	[0.816–8.239]
**Breastfeeding factor**				
Breastfeeding intentions	1.909	6.748	**0.003****	[1.917–23.757]
Nipple shape problems for breastfeeding	-0.839	0.432	0.314	[0.084–2.215]
Breast and nipple troubles at discharge	0.668	1.951	0.231	[0.654–5.823]
**Delivery factor**				
Duration of labor	-0.002	0.998	0.169	[0.996–1.001]
Blood loss at delivery	0.000	1.000	0.926	[0.998–1.002]
Instrumental delivery	-1.279	0.278	0.247	[0.032–2.429]
Use of epidural analgesia in labor	-0.329	0.719	0.781	[0.070–7.375]
**Infant factor**				
Birth weight	0.000	1.000	0.875	[0.999–1.002]
Umbilical cord arterial blood pH value	1.473	4.361	0.794	[0.000–2785.738]
Gestational age at birth	-0.006	0.994	0.871	[0.919–1.074]
Infant gender	-0.568	0.566	0.296	[0.195–1.645]

Supplementary Material 13: Factors influencing the feeding method at 1 month postpartum
The *p*-values were estimated using simple linear regression analysis; *$p<0.05$, **$p<0.01$, ***$p<0.001$
Abbreviations: CI, confidence interval; EPDS, Edinburgh postnatal depression scale.
